# Identification of Three Type II Toxin-Antitoxin Systems in Model Bacterial Plant Pathogen *Dickeya dadantii* 3937

**DOI:** 10.3390/ijms22115932

**Published:** 2021-05-31

**Authors:** Lidia Boss, Marcin Górniak, Alicja Lewańczyk, Joanna Morcinek-Orłowska, Sylwia Barańska, Agnieszka Szalewska-Pałasz

**Affiliations:** 1 Department of Bacterial Molecular Genetics, University of Gdańsk, 80-309 Gdańsk, Poland; a.lewanczyk.825@studms.ug.edu.pl (A.L.); sylwia.baranska@ug.edu.pl (S.B.); agnieszka.szalewska-palasz@ug.edu.pl (A.S.-P.); 2 Department of Molecular Evolution, University of Gdańsk, 80-309 Gdańsk, Poland; marcin.gorniak@ug.edu.pl; 3 Department of Molecular Biology, University of Gdańsk, 80-309 Gdańsk, Poland; joanna.morcinek-orlowska@phdstud.ug.edu.pl

**Keywords:** toxin-antitoxin, *ccdAB*, *ccdAB_2Dda_*, phd-doc, *phd-doc_Dda_*, dhiTA, phytopathogen, soft-rot disease, *Dickeya* *dadantii* 3937

## Abstract

Type II toxin-antitoxin (TA) systems are genetic elements usually encoding two proteins: a stable toxin and an antitoxin, which binds the toxin and neutralizes its toxic effect. The disturbance in the intracellular toxin and antitoxin ratio typically leads to inhibition of bacterial growth or bacterial cell death. Despite the fact that TA modules are widespread in bacteria and archaea, the biological role of these systems is ambiguous. Nevertheless, a number of studies suggests that the TA modules are engaged in such important processes as biofilm formation, stress response or virulence and maintenance of mobile genetic elements. The *Dickeya dadantii* 3937 strain serves as a model for pathogens causing the soft-rot disease in a wide range of angiosperm plants. Until now, several chromosome-encoded type II TA systems were identified in silico in the genome of this economically important bacterium*,* however so far only one of them was experimentally validated. In this study, we investigated three putative type II TA systems in *D. dadantii* 3937: *ccdAB_2Dda_*, *phd-doc_Dda_* and *dhiTA*, which represents a novel toxin/antitoxin superfamily. We provide an experimental proof for their functionality in vivo both in *D. dadantii* and *Escherichia coli*. Finally, we examined the prevalence of those systems across the Pectobacteriaceae family by a phylogenetic analysis.

## 1. Introduction

Soft-rot Pectobacteriaceae (SRP) are Gram-negative, non-spore forming facultative anaerobic bacteria causing soft-rot disease in a wide range of angiosperm plants, limiting the crop yield and quality. SRP include bacteria of two genera: *Pectobacterium,* which are found both in tropical and temperate regions, and *Dickeya*, which were initially thought to be restricted to tropical and subtropical plants and areas. However, since 2005 new genetic clades of *Dickeya* were found in crops and fresh waters in Europe, showing ahigh potential of SRP to adapt to different environmental conditions [1,2,3,4,5,6]. Furthermore, it was suggested that climate change is likely to accelerate evolution and to increase the diversity of bacterial plant pathogens [1,7,8]. Altogether, this makes SRP one of the most scientifically and economically significant group of phytopathogenic bacteria. *Dickeya dadantii* 3937 strain, isolated from an African violet (*Saintpaulia ionantha*), is considered a model organism for the *Dickeya* genus, and thus serves as a subject of extensive research [1,2,9]. Since the genome sequence of *D. dadantii* 3937 was published in 2011 [9], it provided an opportunity to identify and examine chromosome-encoded toxin-antitoxin (TA) systems in this model bacterium. This opportunity is especially fascinating since relatively little is known about those elements in bacterial plant pathogens [10]. The TA systems are intracellular modules consisting of a toxic protein whose activity usually inhibits the host growth, and a cognate antitoxin which neutralizes its toxic effect. Among seven known TA types, one of the best characterized groups is type II TA modules, where the protein antitoxin directly binds and neutralizes the toxin and, furthermore, usually acts as a transcriptional repressor of the TA operon [10,11]. Although TA modules were claimed to be engaged in several different processes and phenomena, including genomic stabilization, abortive phage infection, stress response modulation, virulence and biofilm formation (reviewed in [11,12,13]), their biological role is currently under heated debate [11,12,14,15,16,17]. For instance, a relatively high frequency of toxin-encoding gene deletion with concomitant maintenance of only the cognate antitoxin suggests that chromosomal TA modules function mainly as anti-addiction systems, preventing further TA module invasion, while their role in other processes is rather limited to a particular TA system [15,18]. Nonetheless, it cannot be excluded that the antitoxin maintenance is additionally related to its activity as a transcriptional regulator. Namely, such an orphan antitoxin could possibly regulate *in trans* the genes important for crucial processes or cross-interact with other TA modules present in the bacterial cell [19,20,21]. Moreover, despite the fact that the biological role of TA systems remains elusive in general, a number of data implies that some of them could actually be essential for virulence, biofilm formation and motility in bacterial plant pathogens [19,20,22,23]. For instance, a *Xylella fastidiosa dinJ/relE* knock-out mutant exhibits a hypervirulent phenotype and increased symptoms in grapevine [22], while *Xanthomonas citri* antitoxin-encoding gene *ecnA* deletion affects exopolysaccharide production, motility, oxidative stress response and results in reduced symptom development *in planta* [23]. Considering the inconsistency in the hypotheses of the actual function of TA systems, further studies performed using a bacterial plant pathogen as a model organism may shed new light on TA’s biological role. Thus, the identification and characterization of TA systems in model plant pathogens is urgently needed. Even though several chromosome-encoded type II TA systems were identified in *D. dadantii* 3937 in silico [9]*,* until now only one of them was experimentally validated [10,24]. Nevertheless, the computational analysis does not provide proof of a TA’s systems activity in a living bacterial cell, so it is possible that some of those putative TA modules might be inactive (e.g., due to promoter inactivation or toxin activity impairment) or are simply inaccurately classified [10,14,25]. The aim of this study was to provide an experimental validation of two previously in-silico identified type II TA systems [9]. We also supply experimental proof for the existence of a novel type II TA system in the model bacterium *D. dadantii* 3937.

## 2. Results

### 2.1. Putative TA Systems Identification

The putative type II chromosome-encoded TA systems of *D. dadantii* 3937 selected for investigation in this study were chosen from among TA modules previously predicted in silico and deposited in the NCBI database (*D. dadantii* 3937 NC_014500.1 [9]) or, in the case of DDA3937_RS07415/RS07420 system, predicted by a DNA sequence search with TAfinder [26] and RASTABacteria [27]. The NCBI ID of each gene, respectively, encoding toxin or antitoxin, protein domain pair, the intergenic region length (in bp) and predicted TA family (based on the toxin protein) are shown in Table 1. Putative antitoxins and toxins were classified as CcdA_2Dda_ and CcdB_2Dda_ (DDA3937_RS03880 and DDA3937_RS03885) and Phd_Dda_ and Doc_Dda_ (DDA3937_RS11885 and DDA3937_RS11880), respectively, on the basis of predicted amino acid sequences and protein secondary structure similarity to known and well characterized toxin and antitoxin protein families. No similarities in the predicted amino acid sequences to known proteins were found in the case of the DDA3937_RS07415/RS07420 system, and thus we decided to name this system *dhiTA* (dickeya host inhibition toxin/antitoxin).

**Figure 1:**
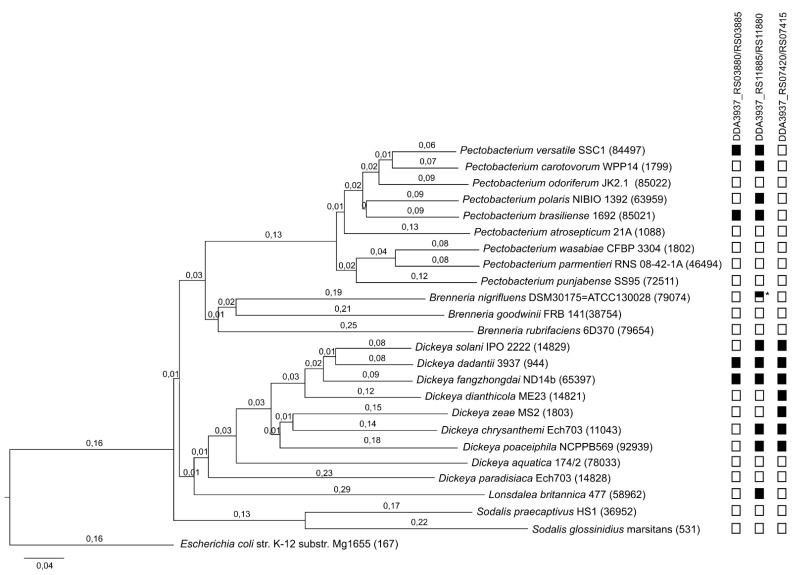
Pectobacteriaceae phylogenetic guide tree based on multiple-genome alignment calculated using Neighbor Joining by the Mauve. Based on the analysis of sequence homology with protein BLAST, the occurrence of individual toxin-antitoxin systems in the analyzed bacterial species was determined. The presence or absence of a given system is marked with a black and white rectangle respectively. * The half-black rectangle indicates the presence of the orphan antitoxin. Branch lengths (shown above branches) represented by a scale bar indicate the number of substitutions per site. The number in the bracket in the taxa name is the genome identifier from NCBI.

To examine prevalence of the three TA systems found in the *D. dadantii* 3937 genome, we analyzed the distribution of each system across the Pectobacteriaceae family. The Pectobacteriaceae phylogenetic guide tree based on the multiple-genome alignment using Mauve indicates that each of the analysed genera forms a separate clade (Figure 1). The topology of the guide tree is similar to pan-genome phylogenetic tree for the genus Pectobacterium [28], maximum likelihood tree based on concatenated partial gene sequences (gyrB, rpoB, atpD and infB) of Brenneria [29] and neighbour-joining tree inferred from the concatenated gene sequences (dnaA, dnaJ, dnaX and recN) of the type or reference strains of Dickeya species [30]. Based on the analysis of the sequence homology done with protein BLAST, the occurrence of individual toxin-antitoxin systems in the analysed bacterial species was determined. The presence or absence of a given system is marked in Figure 1 with a black and white rectangle, respectively. The type II toxin-antitoxin system *ccdAB_2Dda_* (DDA3937_RS03880/RS03885) occurs in two species of the *Dickeya* genus (*D. dadantii* and *D. fangzhongdai*) and in two species of the *Pectobacterium* genus (*P. versatile* and *P. brasiliense*). Type II toxin-antitoxin system *phd-doc_Dda_* (DDA3937_RS11885/RS11880) is the most widespread within the Pectobacteriaceae family. It occurs within the *Pectobacterium*, *Brenneria*, *Dickeya* and *Lonsdalea* genera. In the *Brenneria* genus, part of this system is only present in the *Brenneria nigrifluens* species and is limited only to the antitoxin (indicated with a half-black rectangle in Figure 1). The third of the analysed systems, *dhiTA* (DDA3937_ RS07415/RS07420), was found only in some species of the *Dickeya* genus, forming a clade which indicates an ancestral state of this feature.

**Figure 2:**
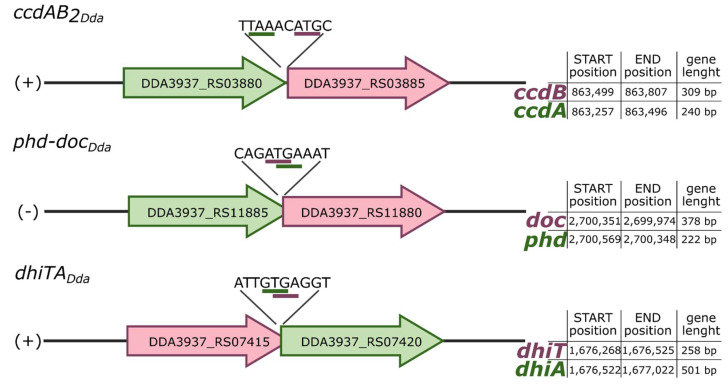
Genetic organization of putative type II toxin-antitoxin (TA) systems predicted in *D. dadantii* 3937. The arrows (red—putative toxins, green—putative antitoxins) indicate the direction of the transcription and do not represent the exact length of the genes. Genomic location of TA systems in the chromosome of *D. dadantii* 3937 and gene length are presented in the tables. The NCBI ID numbers for each putative toxin or antitoxin are depicted in the figure.

**Figure 3:**
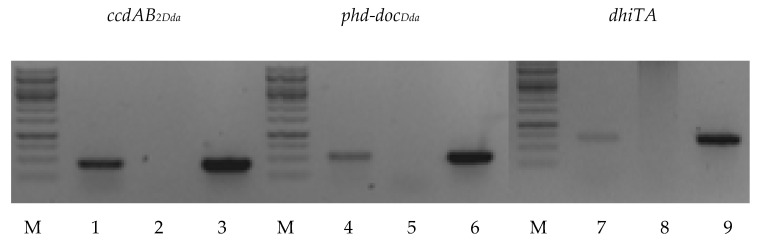
Co-transcription analysis of putative type II toxin-antitoxin (TA) modules in *D. dadantii* 3937. The total RNA was isolated from the logarithmic phase of bacterial growth and used to synthesize cDNAs. Lanes 1, 4 and 7 represent amplification using cDNAs as the template; Lanes 2, 5 and 8 represent the negative control (amplification using RNA without reverse transcriptase reaction); and lanes 3, 6 and 9 represent amplification using genomic DNA (gDNA) as the template. Lane M indicates the O’Gene Ruller DNA marker.

Putative *ccdAB_2Dda_* and *phd-doc_Dda_* systems exhibit typical type II TA operon organization, where the antitoxin gene precedes (and in the case of *phd-doc_Dda_* also overlaps by a few nucleotides the toxin gene), while the gene order of *dhiTA* is switched (Figure 2). To verify whether *D. dadantii* 3937 putative TA modules are co-transcribed, RT-PCR analysis was performed. The total RNA was extracted from *D. dadantii* 3937 and primers complementary to the 3′ end of the coding sequence of each putative TA locus were used to synthetize cDNA. Then, cDNA were employed as a template to perform PCR reaction with the assigned primer pairs (listed in Table S3). The length of obtained DNA fragments was in line with that achieved by amplification using genomic DNA (gDNA) and in correspondence with the length of predicted bicistronic mRNA. No PCR products were detected in negative controls, where PCR was performed using total RNA but without reverse transcriptase reaction, therefore excluding gDNA contamination (Figure 3). The obtained results suggest that all tested genes encoding putative cognate toxins and antitoxins were actively co-transcribed and organized into bicistronic operons.

### 2.2. The Effect of Putative Toxins and Toxin-Antitoxin Complex Overproduction on Bacterial Growth

**Figure 4:**
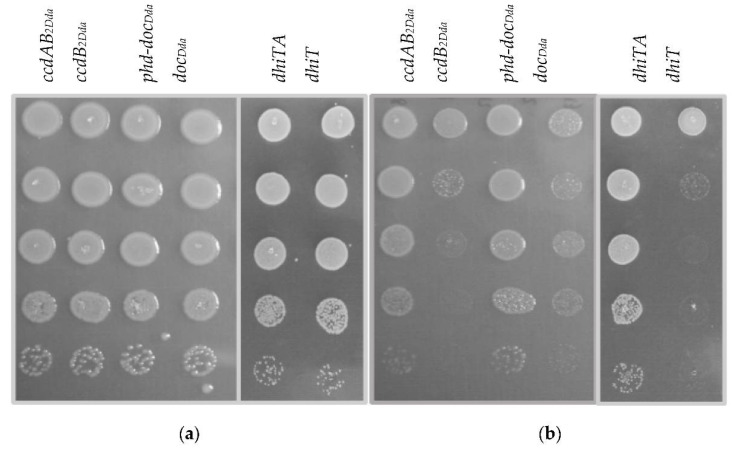
Effect of the putative toxins and toxin-antitoxin complex overexpression on growth of *D. dadantii* 3937. *D. dadantii* 3937 cells, harbouring derivatives of pBAD24 encoding toxin or antitoxin-toxin genes, under control of the *P_BAD_* promoter, were grown to OD_600_ of 0.1–0.15. Each culture was serially diluted, and 15 µL drops of serial dilutions from 1 (top) to 10^−5^ (bottom), were subsequently spotted onto different plates with (**a**) 0.2% D-glucose or (**b**) 0.2% L-arabinose.

**Figure 5:**
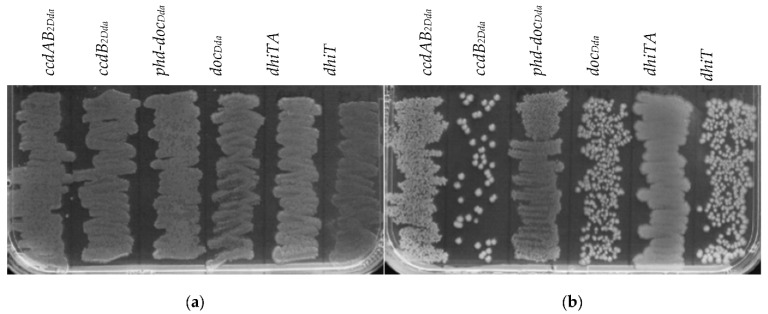
The effect of introducing of the *D. dadantii* 3937 toxins into *E. coli*. *E. coli* cells, harbouring derivatives of pBAD24 encoding the toxin or antitoxin-toxin genes under control of the *P_BAD_* promoter, were grown to OD_600_ of 0.1–0.15. Each culture was streaked onto plates with (**a**) 0.2% D-glucose or (**b**) 0.2% L-arabinose. This experiment was conducted at least 3 times with the same result.

**Figure 6:**
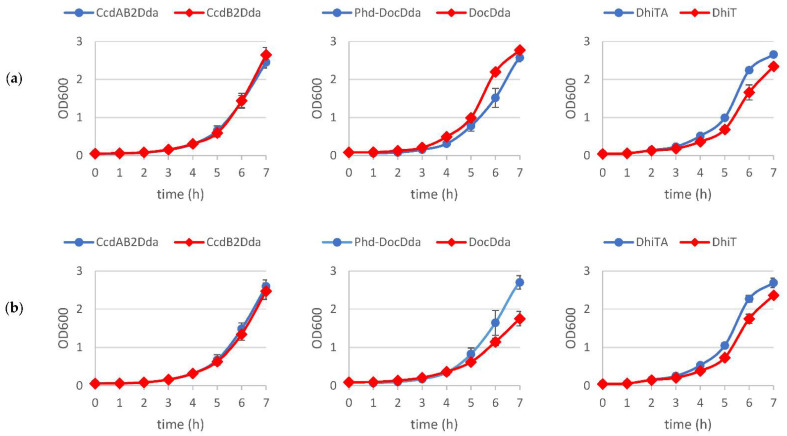
Effect of the induction of expression of the putative toxins on the growth of *D. dadantii* 3937. *D. dadantii* cells, harbouring plasmids encoding the toxin (red ♦) or the antitoxin-toxin (blue ●) genes under control of the *P_BAD_* promoter, were grown to OD_600_ of 0.05–0.08. Each culture was then supplemented with either 0.2% D-glucose (**a**) or 0.2% L-arabinose (**b**). Culture growth was monitored by measuring OD_600_ every hour. The results are an averages of at least 3 independent experiments with SD indicated.

The functioning of type II TA system probably involves oscillation in the toxin and antitoxin level and activities. Typically, both types of proteins are present in the cell in an equilibrium, forming toxin-antitoxin complexes. However, certain factors could affect the cellular toxin-antitoxin ratio, leading to an increase in the free toxin intracellular level. This usually leads to inhibition of bacterial growth or even to bacterial cell death [31]. Likewise, for an active TA system, growth of bacteria ectopically overexpressing toxins is expected to be inhibited, while overproduction of both the toxin and antitoxin would not have a detrimental effect [31,32]. Nevertheless, to observe such a phenomenon, both the toxin and antitoxin intracellular levels have to be strictly controlled and balanced in a way that no free toxin molecules remain unbound [31]. Since putative toxin and antitoxin genes of *D. dadantii* 3937 were shown to be co-transcribed (Figure 3), introduction of the tested genes in their native order under the control of an inducible promoter should ensure balanced toxin and antitoxin intracellular ratio. Thus, to examine the toxicity effect of putative toxins and neutralizing properties of putative antitoxins, we decided to use pBAD24 plasmid derivatives harboring either the toxin-encoding genes or both corresponding genes in their native order. *D. dadantii* 3937 cells, harboring the respective expression vectors were cultured under inducing or repressing conditions in solid and liquid media, and bacterial growth was assessed. The growth on the solid media of *D. dadantii* overexpressing any of the putative toxin genes was inhibited, while overproduction of the toxin-antitoxin complexes did not affect bacterial viability. However, reduction in the colony number of cells overproducing the Doc_Dda_ protein was slightly less pronounced in comparison to other putative toxins (Figure 4). The same experiment was conducted using *Escherichia coli* K-12 MG1655 cells, and similar results were obtained. Overexpression of the putative toxin-antitoxin complexes in *E. coli* did not affect bacterial viability, whereas the toxin overproduction resulted in growth inhibition and this effect was the least evident in case of *doc_Dda_* overexpression (Figure 5). No toxic effect of the Doc_Dda_ was observed in the *E. coli* liquid culture, whereas overexpression of the *dhiT* or *ccdB_2Dda_* resulted in a strong growth inhibition (Figure S1). In contarst, overproduction of the putative Doc_Dda_ or DhiT toxins during growth in liquid medium resulted in a significant reduction in growth rate and prolongation of the generation time of *D. dadantii* cells, while overexpression of *ccdB_2Dda_* did not affect bacterial growth in the liquid medium (Figure 6 and Table S1). Furthermore, to examine the effect of the tested genes’ overexpression on bacterial cell morphology, light microscopy images of *D. dadantii* and *E. coli* cells were acquired and bacterium length was measured from pole to pole with the ImageJ software [33]. Intriguingly, no filament formation was observed during any of the putative toxin-encoding genes overexpression in *D. dadantii* 3937 (Figure S2), whereas strong cells filamentation occurred during *ccdB_2Dda_* overexpression in the *E. coli* cells (Figure S3). Altogether, we demonstrated that all of the tested putative toxins exhibit a toxic effect on both *D. dadantii* and *E. coli* either on the solid medium or both in the solid and liquid culture, while no detrimental effect was observed for overproduction of the toxin-antitoxin complexes. This suggests that the effect of each toxin can be alleviated by its cognate antitoxin and all three putative TA systems are possibly functional both in the *D. dadantii* and *E. coli* cells.

### 2.3. Transcriptional Regulation of the Putative TA Operons

**Figure 7:**
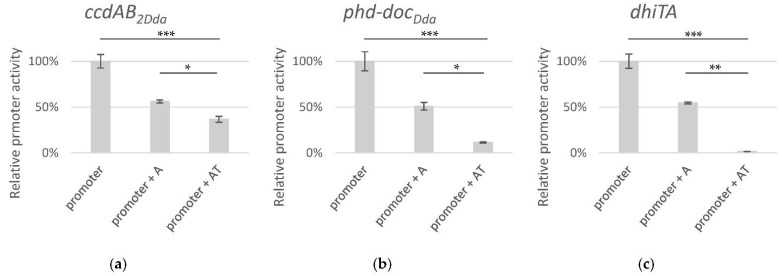
The antitoxin or toxin-antitoxin complex autoregulates the TA operon. (**a**) *ccdAD_2Dda_* system, (**b**) *phd-doc_Dda_* system, (**c**) *dhiTA* system. The pBBRlux plasmid derivatives harbouring transcriptional fusions of promoter regions of different TA operons to the promoter-less *luxCDABE* reporter operon and pBAD24 derivatives harbouring antitoxin or antitoxin-toxin encoding genes were co-transformed into *E. coli* and grown under inducing conditions for 3 h (to the late exponential phase, OD_600_~1.0). Luminescence in RLU (relative light units) was measured. *E. coli* harbouring promoter-less pBBRlux and non-recombinant pBAD24 was used as the negative control. The results represent an average of at least three independent experiments and are shown as a percentage of uninhibited promoter activity, where 100% value corresponds to (**a**) 61,579 +/− 4571 RLU, (**b**) 443 +/− 46 RLU or (**c**) 46,438 +/− 3613 RLU and to 31 +/− 1 RLU for a negative control. * *p* < 0.05, ** *p* < 0.01, *** *p* < 0.001, using ANOVA.

The type II TA genes are organized into operons and their expression is typically negatively regulated at the transcriptional level by the action of the antitoxin alone or in complex with its toxin partner. To assess whether the tested putative TA systems show a similar scheme of regulation, we used pBBRlux-based plasmids, in which a 100 bp DNA fragment encompassing putative promoter regions and start codon of the first gene were inserted upstream of a promoter-less *lux* operon. Since we demonstrated that all tested TA systems are active in *E. coli*, detailed activity assessment was performed in this organism. These transcriptional fusions produced ~60000 RLU for the *ccdAB2_Dda_* putative promoter, ~450 RLU for the *phd-doc_Dda_* putative promoter and ~4500 RLU for the *dhiTA* putative promoter, while the value of ~30 RLU was obtained for a negative control (promoter-less pBBRlux plasmid). In order to examine the influence of cognate antitoxin and toxin-antitoxin complexes on promoter activity *in trans,* suitable pBBRlux-plasmid derivatives and pBAD24-antitoxin or pBAD24-antitoxin-toxin plasmids were co-transformed into *E. coli* MG1655 K-12 strain and grown under inducing conditions for 3 h (to the late exponential phase, OD_600_~1.0). Luminescence was measured, and an inhibitory effect (~50% decrease) of all tested putative antitoxins on their corresponding promoters’ activity was observed (One-way Anova, *p* < 0,001 in each case). Moreover, about a 70% to 99% decrease in promoter activity was observed in the presence of the CcdAB_2Dda_, Phd-Doc_Dda_ or DhiTA toxin-antitoxin complexes (Figure 7) (One-way Anova, *p* < 0.001 in each case). The differences between the promoters’ activity in the presence of the antitoxin and toxin-antitoxin complexes were also observed but with less intensity (one-way Anova, RIR Tukey *post-hoc* test *p* < 0.05 in the cases of the CcdAB_2Dda_ or Phd-Doc_Dda,_ and with *p* < 0.01 in the DhiTA case. These data indicate that the tested promoters’ activity is regulated in a manner characteristic for a canonical type II TA modules.

### 2.4. Plasmid Stability Is Affected by ccdAB_2Dda_ System

**Figure 8:**
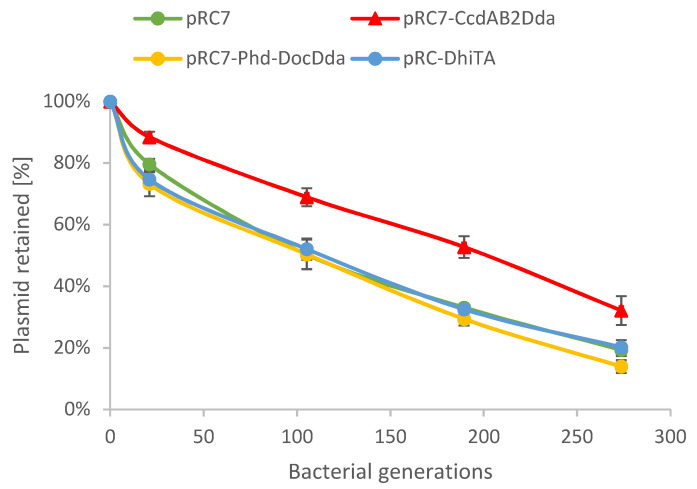
*D. dadantii* 3937 toxin-antitoxin system ccdAB_2Dda_ improves plasmid maintenance in *E. coli* K-12 MG1655*ΔlacZ* strain. Stability assays were conducted with derivatives of the stability probe vector, pRC7: pRC7 does not contain any accessory stability determinants (green), pRC7-ccdAB_2Dda_ contains the *ccdAB_2Dda_* cassette (red), pRC7-phd-doc_Dda_ contains the *phd-doc_Dda_* cassette (yellow), and pRC7-dhiTA contains the *dhiTA* cassette (blue). Generation time corresponds to 17.1 min [39]. Results are an average of at least three independent experiments with SD indicated, and 100% represents the sum of plasmid-harbouring and plasmid-less CFU. To assess statistical significance of the obtained results analysis of covariance (ANCOVA) and HSD Tuckey post-hoc test were applied.

It was shown that some TA systems can promote stability of plasmid maintenance due to toxin-mediated growth inhibition of plasmid-less cells [34,35]. Although this phenomenon is specific for plasmid-encoded TA modules, it has been also reported for several chromosomal TA systems, suggesting that their biological functions could be similar (reviewed in [35]). The CcdAB_2Dda_ and Phd-Doc_Dda_ predicted protein sequences displaysimilarity to proteins of the F plasmid-encoded or P1 phage-encoded TA modules, which were shown to stabilize mobile genetic elements [36,37]. Thus, we decided to test whether any of the *D. dadantii* TA modules contributes to stability ofplasmid maintenance. Therefore, stability assays were conducted with derivatives of pRC7, an unstable mini-F plasmid that is rapidly lost [38]. It carries the *lac+* genes, and therefore its loss is revealed in a chromosomal *Δlac* background by segregation of Lac− clones. We made pRC7 derivatives carrying complete *D. dadantii* TA operons, including promoter regions, and the plasmid-harboring *E. coli* cells were successively subcultured. Samples from each subculture were plated on S-gal supplemented with 1 mM IPTG without antibiotic to obtain single colonies and the black and white colonies were counted. To check the differences in plasmid stability an analysis of covariance (ANCOVA) and HSD Tuckey *post-hoc* test were applied. Statistical analyses show that both the plasmid type and generation time significantly influence the plasmid stability (plasmid type F (3;15) = 10.855, *p* < 0.001), generation time F (1;15) = 759.293, *p* < 0.0001). The HSD Tukey *post-hoc* test shows significant differences between stability of the pRC7-CcdAB_2Dda_ plasmid and the other plasmids (*p* < 0.01). The obtained results indicate that only *ccdAB_2Dda_* increases the plasmid maintenance (Figure 8), suggesting that it could possibly play a role in genomic stabilization or act as an anti-addiction TA system.

## 3. Discussion

The type II toxin-antitoxin systems are one of the best characterized groups of the TA modules, abundant and widespread in many bacteria, including bacterial plant pathogens. Among several methods developed for TA module prediction, genome-based computational approaches are the most popular and successful (reviewed in [10]). The computational approaches are usually based on gene organization and the analysis of amino acid sequence homology of predicted proteins. Thus, such a prediction can be complicated, especially in the case of novel TA superfamily members, and consistently requires experimental validation. An effective technique, called shotgun cloning, was proposed as an alternative method for new TA systems’ identification. Data provided by the use of this method revealed six families of TA systems with no homology to previously characterized modules. Interestingly, two of them were from plant-associated bacteria and four from environmental microbes [10]. Hence, it was suggested that expanding the toxin-antitoxin studies outside of the human pathogen model systems may provide a bigger diversity of TA modules [10]. Moreover, it was already shown that the TA modules can be used for the development of male sterile plants for containment of transgenic plants or for hybrid seed production [40,41]. Furthermore, despite the evolutionary distance, plants can be successfully used as experimental models for human microbial pathogens [42,43,44]. The bacterium species examined in this study, *Dickeya dadantii* 3937, is considered as a model organism for bacterial plant pathogens of the *Dickeya* genus, and therefore, a majority of genetic and virulence studies were performed on this species. Computational analysis of its genome sequence indicated that at least 10 putative type II TA systems could be encoded in the chromosomal DNA of *D. dadantii* 3937 [9]. Nonetheless, until now, only one of them, *ccdAB_Ech_*, was experimentally validated and described [24]. In this study, we present an experimental evidence for in vivo activity of three previously uncharacterized TA systems. These are the *ccdAB_2Dda_* (DDA3937_RS03880/RS03885) and *phd-doc_Dda_* (DDA3937_RS11885/RS11880), that were formerly identified in silico by the gene prediction method using protein homology analysis [9], and *dhiTA* (DDA3937_ RS07415/RS07420), which was identified in this work as a member of a potential novel TA superfamily. We found no homology between the predicted DhiTA proteins’ sequences to any known TA proteins. Data provided by phylogenetic analysis of the Pectobacteriaceae family imply that the *dhiTA* module has probably appeared for the first time in the genome of the common ancestor of seven *Dickeya* species and is conserved within the descendants of this species (*D. dadantii, D. solani, D. fangzhongdai, D. dianthicola, D. zeae, D. chrysathemi, D. poceiphila*) (Figure 1). Thus, we propose that this system represents a new toxin superfamily, DhiT, and a new antitoxin superfamily, DhiA (dickeya host inhibition toxin/antitoxin).

We demonstrated that all putative TA genes tested in this work were organised into bicistronic operons and actively co-transcribed. Subsequently, we showed that overproduction of any putative toxin results in reduction in bacterial growth both in *D. dadantii* 3937 and *E. coli* K-12 MG1655, while simultaneous overexpression of each putative toxin and its cognate antitoxin did not have the detrimental effect. This suggests that all candidates may function as TA systems. However, a similar outcome may be observed in overexpression of genes encoding components of the restriction-modification systems (RM) or toxin-immunity pairs. Effectors of the toxin-immunity pairs are a group of proteins secreted into the environment and they act on competitive bacteria lacking the immunity proteins. Hence, this kind of toxin typically has an N-terminal secretion signal [45,46]. Nevertheless, it is not likely that any of the tested candidates encodes a toxin-immunity pair, because none of the analyzed predicted toxins bear putative N-terminal signal peptide (data not shown). The second group of potentially toxic gene modules, the type II RM systems, encodes a toxic restriction enzyme and a modification enzyme, serving as an antitoxin. Similarly to TA modules, the modification enzyme may also act as a transcriptional regulator of the RM operon. Nonetheless, in contrast to the RM systems, transcriptional regulation of the canonical type II TA system involves the activity of the toxin as a co-repressor [47,48]. Therefore, to exclude the possibility that any of the tested operons encodes an RM system, we provide evidence that the *ccdAB_2Dda_*, *phd-doc_Dda_* and *dhiTA* systems exhibit transcriptional regulation pattern specific for the canonical type II TA system, with the toxin working as a co-repressor. Interestingly, the uninhibited activity of the *phd-doc_Dda_* promoter was noticeably lower than the activity of the other tested promoters, suggesting either partial inactivation of this module or requirement of a specific conditions for its activity. Phylogenetic analysis indicated that, although *phd-doc_Dda_* is the most widespread within the Pectobacteriaceae family among all examined systems, that the loss of these genesby deletion was independent in different species. Proteins homologous to Phd-Doc_Dda_ occur within the *Pectobacterium*, *Dickeya* and *Lonsdaela* genera, while in the *Brenneria* genus only the antitoxin of this system is present in the *Brenneria nigrifluens* species. Moreover, we observed that growth inhibition during Doc_Dda_ overproduction was less pronounced in comparison to the effect of *ccdB_2Dda_* or *dhiT* overexpression. On the other hand, overexpression of *doc_Dda_* in liquid cultures resulted in an evident reduction in *D. dadantii* 3937 growth rate, but did not affect *E. coli* growth. The basis of this phenomenon is ambiguous for the moment and requires further investigation.

According to the results of the phylogenetic analysis, the *ccdAB_2Dda_* system occurs in two species of the *Dickeya* genus (*D. dadantii and D. fangzhongdai*) and in two species of the *Pectobacterium* genus (*P. versatile* and *P. brasiliense*). This indicates that its appearance is probably due to horizontal gene transfer. The CcdB_2Dda_ toxin displays a certain level of similarity to the *E. coli* F-plasmid-carried CcdB and chromosomally encoded CcdB_Ech_ toxin of *D. dadantii 3937*. Both, the *ccdAB* and *ccdAB_Ech_* systems confer plasmid stability [24,36], and as our results suggested, *ccdAB_2Dda_* also possesses a similar activity, although the effect of plasmid maintenance stabilization was obscure. Likewise, CcdB and CcdB_Ech_ were both shown to poison gyrase [24,43,44,45,46]. Overexpression of *ccdB* causes, among other effects, reduced DNA synthesis, activation of the SOS regulon and cell filamentation [49,50,51,52]. Overproduction of CcdB_2Dda_ toxin resulted in an evident reduction in colony number in *D. dadantii* 3937 when cultured on solid medium, while it did not significantly affect bacterial growth rate in liquid cultures. Surprisingly, *D. dadantii* 3937 overexpressing *ccdB_2Dda_* exhibits regular cell morphology, while overproduction of CcdB_2Dda_ in *E. coli* resulted in strong filamentation and reduction of growth rate in liquid culture. We suppose that no filament formation in *D. dadantii* 3937 overexpressing *ccdB_2Dda_* could be connected either to the presence of the chromosomally encoded copy of the *ccdAB_2Dda_* within the cells or to the cross-regulation between *ccdAB_2Dda_* and other TA coexisting in this bacterial genome, such as *ccdAB_Ech_*. However, so far there is little evidence for this type of interaction between multiple TA paralogs encoded by a single bacterial chromosome, especially in Gram-negative bacteria (reviewed in [21,53]). Further experiments are needed to elucidate whether homologous and non-homologous TA systems of *D. dadantii* 3937 can affect the activity of each other.

To conclude, we present three actively transcribed, functional chromosomally encoded type II TA systems in a model organism *D. dadantii* 3937. We believe that expanding the knowledge about TA systems in plant-related bacteria may help not only to expand diversity of the TA systems, but also improve understanding of their biological role and allow taking advantage of their biotechnological potential as well.

## 4. Materials and Methods

### 4.1. Bacterial Strains, Plasmids, Primers, and Growth Conditions

Bacterial strains and plasmids used in this study are listed in Table S1. Primers are listed in Table S2. The *D. dadatii* 3937 strain was cultured in Tryptic Soy Broth (TSB) or on Tryptic Soy Agar (TSA), at 30 °C. *E. coli* strains were cultured in TSB or Luria-Bertani (LB) broth or on TSA or LB agar at 30 or 37 °C, as indicated. When required, antibiotics were added to the following final concentrations: 50 µg/mL ampicillin or 34 µg/mL chloramphenicol. Molecular cloning was performed by Gibson–Assembly method [54] using pBAD24 plasmid digested with SmaI or pRC7 plasmid digested with ApaI enzyme and DNA fragments amplified as indicated in Table S3. For promoter activity assay, the 100 bp upstream region, preceding each antitoxin start codon (or the toxin start codon in case of the *dhiTA* system), was selected as a promoter regions using BPROM [55]. The promoter region DNA fragments were PCR-amplified and cloned into pBBRlux plasmid [56] digested with BamHI enzyme. All plasmids used were introduced into *D. dadantii* 3937 strain by electroporation: cells were electroporated with 50 ng plasmid DNA in a 0.4 mm gap electroporation cuvette at 1.7 kV.

### 4.2. Bioinformatics and Statistical Analysis

The putative type II TA systems in *D. dadantii* 3937 were identified on the basis of annotations obtained from DNA sequences deposited in the NCBI database (*D. dadantii* 3937 NC_014500.1 [9]) or predicted with the TA finder, Shanghai, China (https://db-mml.sjtu.edu.cn/TAfinder/index.php, 26 June 2019) and RASTABacteria, Rennes, France [27]. The 250 bp upstream DNA sequence fragments preceding the predicted toxins and antitoxins encoding genes were analysed with BPROM [55], in order to find putative promoter regions. Predicted toxin amino acid sequences were analysed with SignalP 5.0, Lyngby, Denmark [57], in order to find putative N-terminal signal peptides. Statistical analyses were performed using Statistica 13.1. All analyses were performed with the alpha level set at 0.05.

### 4.3. Phylogenetic Analysis

23. fully annotated whole genomes (chromosomal level) of members of the Pectobacteriaceae family were searched with the Protein BLAST algorithm (NCBI), Bethesda, Maryland, USA to find homologous sequences of analysed toxin-antitoxin systems (DDA3937_RS03880/RS03885, DDA3937_RS11885/RS11880, DDA3937_RS07420/RS07415). MAFFT, Osaka, Japan [58] server was used to align amino acid sequences. All matrices were analysed using PAUP (Phylogenetic Analysis Using Parsimony and Other Methods) version 4.0a [59]. The optimality criterion was set to p-distance using the Neighbour-Joining algorithm (NJ). The robustness of the tree topology was assessed by bootstrap analyses based on 1000 replicates. To estimate average evolutionary divergence over sequence pairs within genus and between genera the JTT matrix-based model [60] with a gamma distribution was used, using the MEGA X software [61]. To determine whether the presence or absence of a given toxin-antitoxin system is correlated with phylogenetic relationship the progressive genome alignment according to a guide tree was built up after computing a pairwise genome content distance using the neighbor joining method and a pairwise breakpoint distance matrix using Mauve algorithm [62]. *RNA Isolation and RT-PCR Analysis*

*D. dadantii* 3937 was grown to OD_600 of_ 0.5–0.6 and used to extract total RNA. Total RNA was purified using an RNA isolation RNAmini Kit (A&A Biotechnology, Poland), according to the manufacturer’s protocol. DNA was digested using TURBO DNA free Kit™ (Ambion, USA), according to the manufacturer’s protocol. The RNA integrity and concentration were determined by agarose gel electrophoresis and NanoDrop, respectively. The cDNAs were generated from these RNA samples with TranScriba Kit (A&A Biotechnology, Poland). We used specific primers listed in Table S3 to confirm the co-transcription of putative toxin and antitoxin genes.

### 4.4. TA Systems Toxicity Assay (Solid Medium)

*D. dadantii* 3937 cells, harbouring the pBAD24-toxin or pBAD24-antitoxin-toxin constructs, were incubated in TSB broth, supplemented with 50 µg/mL ampicillin and 0.2% D-glucose to OD_600_ of 0.1–0.15. Each culture was serially diluted, and 15 µL drops of the relevant dilutions (from of 1 to 10^−5^), were successively spotted onto different plates, one supplemented with 0.2% L-arabinose (induction of tested genes expression), and one supplemented with 0.2% D-glucose (repression conditions), serving as a control. The same procedure was conducted using *E. coli* K-12 MG1655 strain. Each experimental procedure was performed at least two times with at least three biological replicates.

### 4.5. TA Systems Toxicity Assay (Liquid Medium)

*D. dadantii* 3937 cells, harbouring the pBAD24-toxin or pBAD24-antitoxin-toxin plasmid were grown in TSB broth, with an additional 50 µg/mL ampicillin and 0.2% D-glucose at 30 °C overnight. The next day, the cultures were diluted 1:100 into fresh medium, supplemented with 50 µg/mL ampicillin (TSB-ampicillin), and grown to OD_600_ of 0.05–0.08. Each culture was then divided into two parts. One half was grown in the presence of 0.2% D-glucose (repression conditions), while the other was grown in the presence of 0.2% L-arabinose (induction conditions). Culture growth was monitored by measuring OD_600_ every hour. The same procedure was conducted using *E. coli* K-12 MG1655 strain. Each experimental procedure was performed at least two times with at least three biological replicates. During growth experiments, samples of *D. dadantii* 3937, harbouring the related plasmids under inducing or repressing conditions, were collected at the 3 h time point, and light microscopy (LeicaDMI4000 microscope) images were acquired using a 100× objective, under oil-immersion. Bacterium length was measured from pole to pole with the ImageJ software [33]. Cell length was measured for 100 bacteria for each of the tested cultures as described in [63,64]. The same procedure was conducted using the *E. coli* K-12 MG1655 strain.

### 4.6. Promoter Activity Assay

The pBBRlux-based plasmids and corresponding pBAD24-antitoxin or pBAD24-antitoxin-toxin plasmids were co-transformed into the *E. coli* MG1655 K-12 strain. Overnight cultures carrying recombinant plasmids were diluted (1:100) into fresh TSB medium supplemented with ampicillin, chloramphenicol and 0.2% arabinose (inducing conditions), and grown 3 h at 30 °C (to the late exponential phase, OD_600_~1.0). Then luminescence of 200 µL of cells was measured in a luminometer (Berthold Technologies, Junior) as previously described [65]. The promoter-less luxCDABE-fusion vector pBBRlux was used as a negative control. Results in relative light units (RLU) were shown as a percentage of uninhibited promoter activity.

### 4.7. Plasmid Stability Assay

The pRC7-based plasmids were transformed into the *E. coli* MG1655*ΔlacZ* strain and non-recombinant pRC7 plasmid was used as a control. Bacteria containing different constructs were grown overnight at 37 °C under selective conditions. 1 µL of the resulting culture was used to inoculate 10 mL of fresh TSB medium again with antibiotic pressure and left to grow with shaking for 6 h. Next, 1:10,000 dilutions were made every 6 and 18 h in fresh medium without the selective pressure. Successive subcultures were repeated 8 times. Samples from each subculture were plated on S-gal supplemented with 1 mM IPTG without antibiotic to obtain single colonies. White and black colonies were counted after overnight incubation at 37 °C.

## Figures and Tables

**Table 1:** Putative type II toxin-antitoxin (TA) systems predicted in *D. dadantii* 3937.

TA Name	Toxin (T) NCBI ID [9]	Antitoxin (A) NCBI ID [9]	Strand	Distance (bp) ^1^	Domain Pair ^2^	T/A Family
*ccdAB_2Dda_*	DDA3937_RS03885	DDA3937_RS03880	+	+3	MazF-like domain/RHH-like domain	ccdB/ccdA
*phd-doc_Dda_*	DDA3937_RS11880	DDA3937_RS11885	−	−3	Fic-like domain/PHD-like domain	Doc/Phd/YefM
*dhiTA*	DDA3937_RS07415	DDA3937_RS07420	+	−3	DUF4160/DUF2442	NH

^1^ The distance (bp) indicates the physical distance (in bp) between the putative antitoxin and toxin coding sequences, and the toxin and antitoxin genes overlapping (−) or separation (+) by a few nucleotides; ^2^ A domain pair represents the TA protein domain pair of each antitoxin and its cognate toxin. NH, no homology to known toxin and antitoxin found.

## Data Availability

Not applicable.

## Supplementary Materials

The following are available online at https://www.mdpi.com/article/10.3390/ijms22115932/s1.
