# Supplementary material for: Identification of Three Type II Toxin-Antitoxin Systems in Model Bacterial Plant Pathogen Dickeya dadantii 3937
Source: Int J Mol Sci. 2021 May 31;22(11):5932. doi: 10.3390/ijms22115932 (PMC8198452; doi:10.3390/ijms22115932)
Supplement: Supplementary file 1 [file ijms-22-05932-s001.zip › ijms-1241183-supplementary.pdf]

Article

Identification of Three Type II Toxin-Antitoxin Systems in Model Bacterial Plant Pathogen *Dickeya dadantii* 3937

Lidia Boss [1,*], Marcin Górniak [2], Alicja Lewańczyk [1], Joanna Morcinek-Orłowska [3], Sylwia Barańska [1] and Agnieszka Szalewska-Pałasz [1]

1 Department of Bacterial Molecular Genetics, University of Gdańsk,80-309 Gdańsk, Poland; a.lewanczyk.825@studms.ug.edu.pl (A.L.); sylwia.baranska@ug.edu.pl (S.B.); agnieszka.szalewska-palasz@ug.edu.pl (A.S.-P.)
2 Department of Molecular Evolution, University of Gdańsk, 80-309 Gdańsk, Poland; marcin.gorniak@ug.edu.pl
3 Department of Molecular Biology, University of Gdańsk, 80-309 Gdańsk, Poland; joanna.morcinek-orlowska@phdstud.ug.edu.pl (J. M.-O.)
* Correspondence: lidia.boss@ug.edu.pl

Supplementary Data

Table S1. Generation time (min) of the *D. dadantii* 3937 overexpressing the putative toxins or toxin-antitoxin complexes.

	pBAD-toxin	pBAD-antitoxin-toxin
ccdAB$_{2Dda}$	63.5 +/- 2.4	62.4 +/-7.9
phd-doc$_{2Dda}$	83.2 +/- 3.9	56.5 +/-2.8
dhiTA	78.9 +/-2.9	65.9 +/-1.5

Table S2. Bacterial strains and plasmids used in this study.

Strain or plasmid	Relevant characteristics	Source or reference
**Strains**		
***Dickeya dadantii* 3937**	Genomic DNA and total RNA source, host for in vivo experiments with selective expression vectors	Wild-type strain isolated from *Saintpaulia ionantha* [63]
Escherichia coli		
DH5α	Cloning host for recombinant vectors	[64]
MG1655	Host for promoter activity assays and for in vivo experiments with selective expression vectors	[65]
MG1655*ΔlacZ*	Host for plasmid stabilization assay	Lab stock
**Plasmids**		
pBAD24	Selective expression vector; amp^R	[66]
pBAD24-ccdAB$_{2Dda}$	pBAD24 derivative containing *ccdAB*$_{2Dda}$ genes under control of arabinose-inducible P_{BAD} promoter	This study
pBAD24-ccdA$_{2Dda}$	pBAD24 derivative containing *ccdA*$_{2Dda}$ gene under control of arabinose-inducible P_{BAD} promoter	This study
pBAD24-ccdB$_{2Dda}$	pBAD24 derivative containing *ccdB*$_{2Dda}$ gene under control of arabinose-inducible P_{BAD} promoter	This study
pBAD24-phd-doc$_{Dda}$	pBAD24 derivative containing *phd-doc*$_{Dda}$ genes under control of arabinose-inducible P_{BAD} promoter	This study
pBAD24-phd$_{Dda}$	pBAD24 derivative containing *phd*$_{Dda}$ gene under control of arabinose-inducible P_{BAD} promoter	This study

Strain or plasmid	Relevant characteristics	Source or reference
pBAD24-doc$_{Dda}$	pBAD24 derivative containing *doc*$_{Dda}$ gene under control of arabinose-inducible P_{BAD} promoter	This study
p24dhiTA	pBAD24 derivative containing *dhiTA* genes under control of arabinose-inducible P_{BAD} promoter	This study
p24dhiA	pBAD24 derivative containing *dhiA* gene under control of arabinose-inducible P_{BAD} promoter	This study
p24dhiT	pBAD24 derivative containing *dhiT* gene under control of arabinose-inducible P_{BAD} promoter	This study
pBBRlux	Vector for generating transcriptional fusion to *lux*, Cm^r	[55]
pLuxccdAB$_{2Dda}$	pBBRlux derivative containing the 100bp fragment upstream *ccdA*$_{2Dda}$ gene, cloned upstream promoter-less *luxCDABE operon*	This study
pLuxphd-doc$_{2Dda}$	pBBRlux derivative containing the 100bp fragment upstream *phd*$_{Dda}$ gene, cloned upstream promoter-less *luxCDABE operon*	This study
pLuxdhiTA	pBBRlux derivative containing the 100bp fragment upstream *dhiT* gene, cloned upstream promoter-less *luxCDABE operon*	This study
pRC7	Unstable, low-copy-number, mini-F derivative of pFZY1, used for plasmid stabilization assays	[35]
pRC7-ccdAB$_{2Dda}$	pRC7 derivative containing *ccdAB*$_{2Dda}$ toxin-antitoxin operon including promoter region, cloned within ApaI site	This study
pRC7-phd-doc$_{Dda}$	pRC7 derivative containing *phd-doc*$_{Dda}$ toxin-antitoxin operon including promoter region, cloned within ApaI site	This study
pRC7-dhiTA	pRC7 derivative containing *dhiTA* toxin-antitoxin operon including promoter region, cloned within ApaI site	This study

Table S3. Primers used in this study.

Primer	Sequence (5′-3′)	Relevant characteristics
1	GCTAGCAGGAGGAATTCACCATGCCGACTACAAAAGCATACGA	Primer (forward) for *ccdA(B)*$_{2Dda}$ amplification with primer 2 or 4, fragment cloned into pBAD24 digested with SmaI
2	GTCGACTCTAGAGGATCCCCTTAAAACGTCCTGAATCAT	Primer (reverse) for *ccdA*$_{2Dda}$ amplification with primer 1, fragment cloned into pBAD24 digested with SmaI
3	GCTAGCAGGAGGAATTCACCATGCAGTACATGGTGTACG	Primer (forward) for *ccdB*$_{2Dda}$ amplification with primer 4, fragment cloned into pBAD24 digested with SmaI
4	GTCGACTCTAGAGGATCCCCGGGTCAAAACCCGTCTAGCAAAA	Primer (reverse) for *ccdA(B)*$_{2Dda}$ amplification with primer 1 or 3, fragment cloned into pBAD24 digested with SmaI
5	GCTAGCAGGAGGAATTCACCATGAAGACTTATACC	Primer (forward) for *phd(doc)*$_{Dda}$ amplification with primer 6 or 8, fragment cloned into pBAD24 digested with SmaI
6	GTCGACTCTAGAGATCCCCTCATCTGTCGGCGAG	Primer (reverse) for *phd*$_{Dda}$ amplification with primer 5, fragment cloned into pBAD24 digested with SmaI
7	GCTAGCAGGAGGAATTCACCATGAAATGGGTGAGTGCGC	Primer (forward) for *doc*$_{Dda}$ amplification with primer 8, fragment cloned into pBAD24 digested with SmaI

Primer	Sequence (5′-3′)	Relevant characteristics
8	GTCGACTCTAGAGGATCCCCTTATTTTTCCACCCGTTG	Primer (reverse) for *phd(doc)*$_{Dda}$ amplification with primer 5 or 7, fragment cloned into pBAD24 digested with SmaI
9	GCTAGCAGGAGGAATTCACCATGCCTGAAATTGAC	Primer (forward) for *dhiT*(*A*) amplification with primer 10 or 12, fragment cloned into pBAD24 digested with SmaI
10	GTCGACTCTAGAGGATCCCCTCACAATTTTCCCGGATG	Primer (reverse) for *dhiT* amplification with primer 10, fragment cloned into pBAD24 digested with SmaI
11	GCTAGCAGGAGGAATTCACCATGCATAAAATCATC	Primer (forward) for *dhiA* amplification with primer 12, fragment cloned into pBAD24 digested with SmaI
12	GTCGACTCTAGAGGATCCCCCTACTTCTCTTCCAG	Primer (reverse) for *dhiT(A)* amplification with primer 10 or 11, fragment cloned into pBAD24 digested with SmaI
13	GGTGGCGGCCGCTCTAGAACTAGTGCCTGAGCGGGTATCG CGGCG	Primer (forward) for putative promoter region of *ccdAB*$_{2Dda}$ amplification with primer 14, fragment cloned into pBBRlux digested with BamHI
14	ATCCATTTTGCGGCCGCAACTAGAGGTTGCCCTCCATTTAC ACGT	Primer (reverse) for putative promoter region of *ccdAB*$_{2Dda}$ amplification with primer 13, fragment cloned into pBBRlux digested with BamHI
15	GGTGGCGGCCGCTCTAGAACTAGTGGGTTGGGGGCTTTTTT TATA	Primer (forward) for putative promoter region of *phd-doc*$_{Dda}$ amplification with primer 16, fragment cloned into pBBRlux digested with BamHI
16	ATCCATTTTGCGGCCGCAACTAGAGGGCTCCCCCTTGAAAT GTAC	Primer (reverse) for putative promoter region of *phd-doc*$_{Dda}$ amplification with primer 15, fragment cloned into pBBRlux digested with BamHI
17	GGTGGCGGCCGCTCTAGAACTAGTGGTGGAATCAACATGA AACCT	Primer (forward) for putative promoter region of *dhiTA* amplification with primer 18, fragment cloned into pBBRlux digested with BamHI
18	ATCCATTTTGCGGCCGCAACTAGAGTGGCACTCCTTGTCAA TGAG	Primer (reverse) for putative promoter region of *dhiTA* amplification with primer 17, fragment cloned into pBBRlux digested with BamHI
19	TCACCAGCAAATCGCGCTGTTAGCGTGAGCGGGTATCGCG GCGTA	Primer (reverse) for *ccdAB*$_{2Dda}$ amplification, including putative promoter region, used with primer 20, fragment cloned into pRC7 digested with ApaI
20	AGACGCGCCGAGACAGAACTTAATGTCAAAACCCGTCTAG CAAAA	Primer (forward) for *ccdAB*$_{2Dda}$ amplification, including putative promoter region, used with primer 19, fragment cloned into pRC7 digested with ApaI
21	TCACCAGCAAATCGCGCTGTTAGCGTTGGGGGCTTTTTTAT AAC	Primer (reverse) for *phd-doc*$_{Dda}$ amplification, including putative promoter region, used with primer 22, fragment cloned into pRC7 digested with ApaI

Primer	Sequence (5′-3′)	Relevant characteristics
22	AGACGCGCCGAGACAGAACTTAATGTTATTTTTCCACCCGT TGTC	Primer (forward) for *phd-doc*$_{Dda}$ amplification, including putative promoter region, used with primer 21, fragment cloned into pRC7 digested with ApaI
23	TCACCAGCAAATCGCGCTGTTAGCGTGGAATCAACATGAA ACCTT	Primer (forward) for *dhiTA* amplification, including putative promoter region, used with primer 24, fragment cloned into pRC7 digested with ApaI
24	AGACGCGCCGAGACAGAACTTAATGCTACTTCTCTTCCAGT TGTA	Primer (reverse) for *dhiTA* amplification, including putative promoter region, used with primer 23, fragment cloned into pRC7 digested with ApaI
25	ACTACAAGCATACGACAC	Primer (forward) for RT-PCR analysis of *ccdAB*$_{2Dda}$ module transcript
26	TCATCACCAGATAATCAC	Primer (reverse) for RT-PCR analysis of *ccdAB*$_{2Dda}$ module transcript
27	TATACCATTACTGAAGCC	Primer (forward) for RT-PCR analysis of *phd-doc*$_{Dda}$ module transcript
28	CTTAATGGTGAGTTCTTC	Primer (reverse) for RT-PCR analysis of *phd-doc*$_{Dda}$ module transcript
29	GATGATGAGTTGATCATC	Primer (forward) for RT-PCR analysis of *dhiTA* module transcript
30	CTCTTCCAGTTGTAACAC	Primer (reverse) for RT-PCR analysis of *dhiTA* module transcript

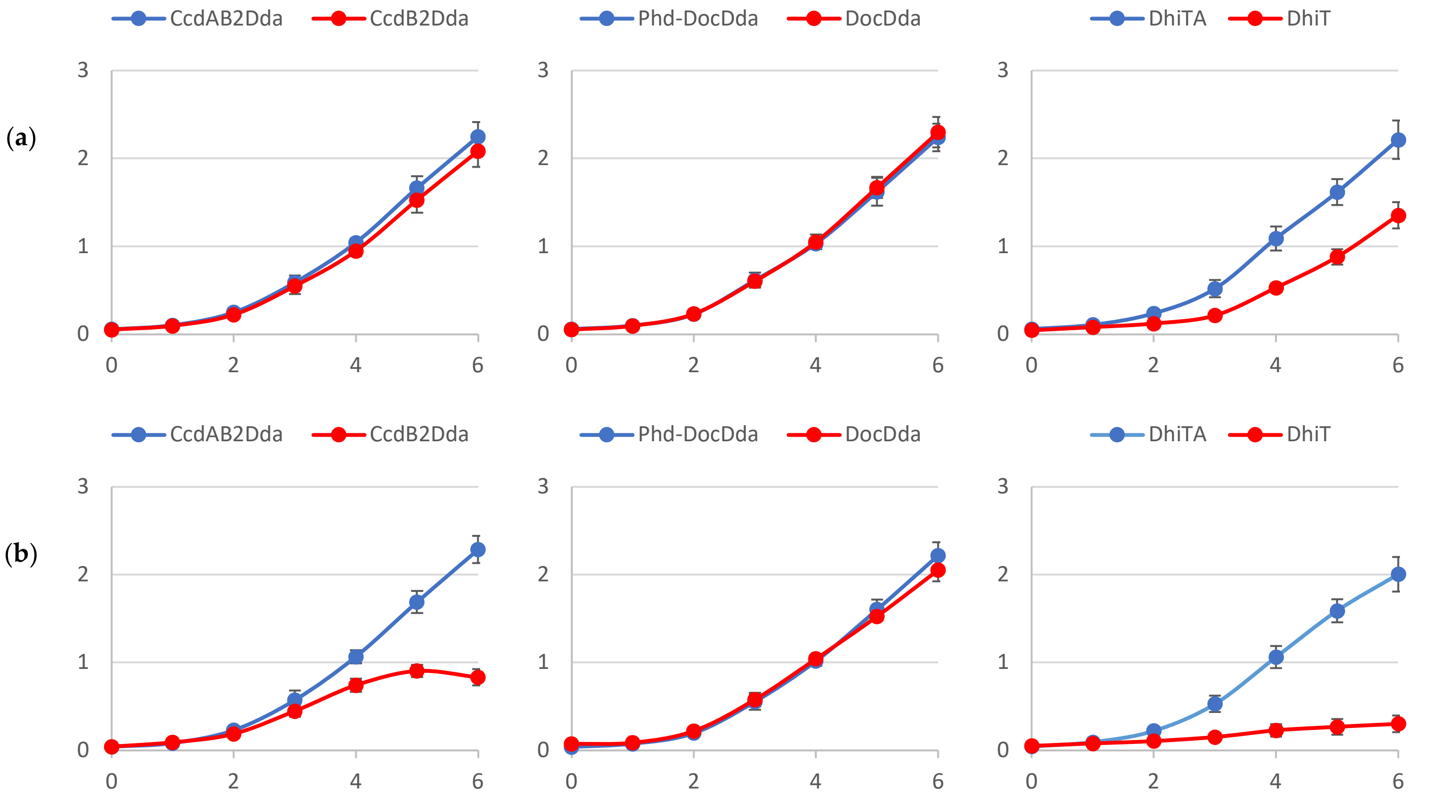

Figure S1. Effect of induction of expression of the putative toxins on growth of the *E.* coli K-12 MG1655. *E. coli* cells, harbouring plasmids encoding the toxin (red ♦) or antitoxin-toxin (blue •) genes under control of the P_{BAD} promoter, were grown to OD_{600} of 0.05–0.08. Each culture was then supplemented with either 0.2 % D-glucose (**a**) or 0.2 % L-arabinose (**b**). Culture growth was monitored by measuring OD_{600} every hour. The results are an averages of at least 3 independent experiments with SD indicated.

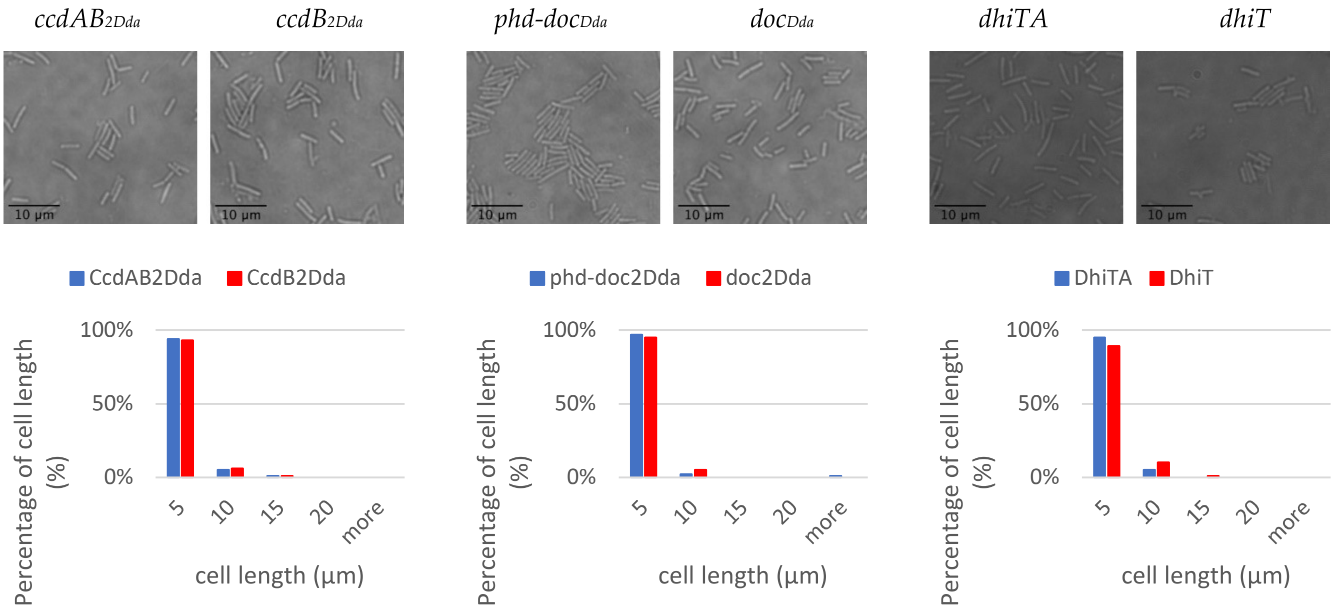

(**a**) (**b**) (**c**)

Figure S2. The effect of overexpression of the toxins on the *D. dadantii* 3937 cell morphology. pBAD24 derivatives harbouring genes encoding (**a**) CcdAB$_{2Dda}$ system or toxin CcdB$_{2Dda}$, (**b**) Phd-Doc$_{Dda}$ system or Doc$_{Dda}$, toxin (**c**) DhiTA system or DhiT toxin were introduced into *D. dadantii* 3937 cells and were induced with 0.2 % arabinose. *D. dadantii* 3937 cells cultured in the same conditions were used as a cell length control. Light microscope morphology of *D. dadantii* 3937 cells (×100), cell length was measured for 100 bacteria for each strain. Examples of relevant microphotographs are above the graphs.

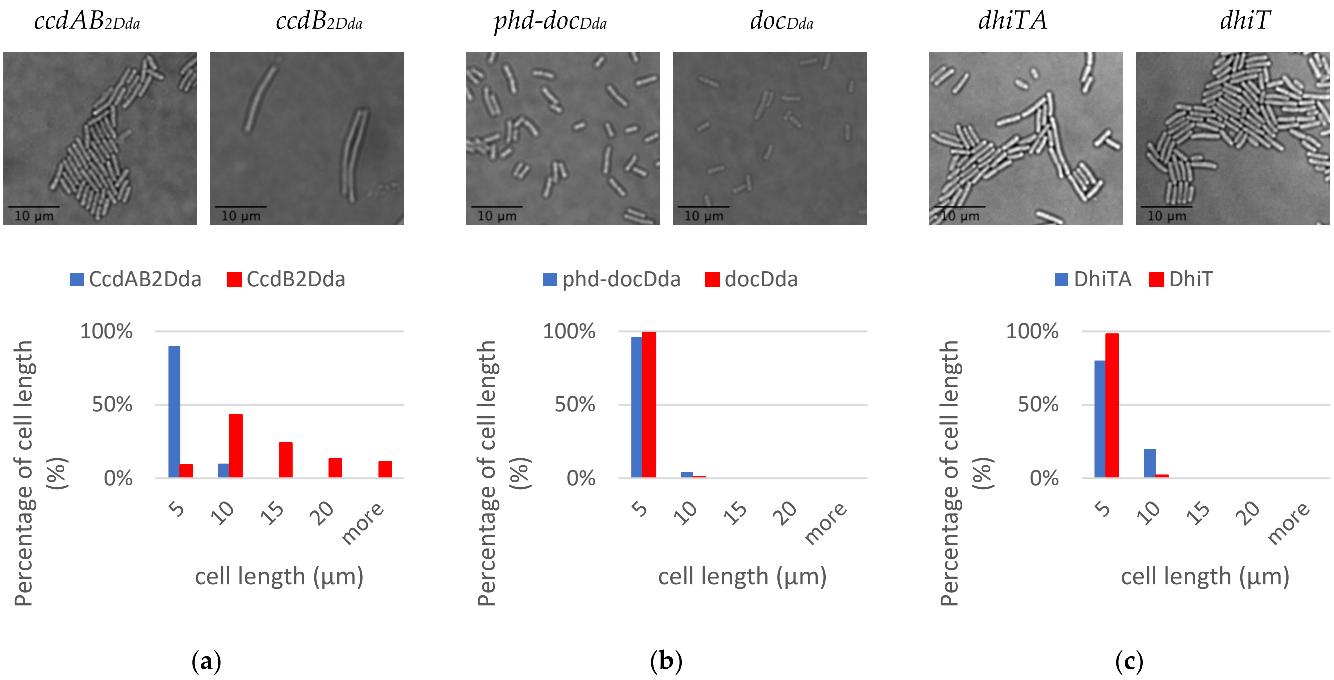

Figure S3. The effect of overexpression of the *D. dadantii* 3937 toxins on the *E. coli* cell morphology. pBAD24 derivatives harbouring genes encoding (**a**) CcdAB$_{2Dda}$ system or toxin CcdB$_{2Dda}$, (**b**) Phd-Doc$_{Dda}$ system or Doc$_{Dda}$ toxin (**c**) DhiTA system or DhiT toxin were introduced into *E. coli* cells and were induced with 0.2 % arabinose. *E. coli* cells cultured in the same conditions were used as a cell length control. Light microscope morphology of *E. coli* cells (×100), cell length was measured for 100 bacteria for each strain. Examples of relevant microphotographs are above the graphs.
